# Development and validation of a screening method for difficult tracheal intubation based on geometric simulation and computer technology

**DOI:** 10.1186/s12871-023-02312-9

**Published:** 2023-10-25

**Authors:** Yue Yu, Jingjing Cao, Xinyuan Tang, Zhiyuan Dong, Jianling Xu, Bin Wang, Pingping Cheng, Mingfang Wang, Yue Wu, Weidong Yao, Xiaogan Jiang

**Affiliations:** 1 Department of Anesthesiology, The First Affiliated Hospital of Wannan Medical College, Wuhu, Anhui China; 2 Department of Anesthesiology, The First People’s Hospital of Wuhu City, Wuhu, Anhui China; 3 Anhui Province Clinical Research Center for Critical Care Medicine (Respiratory Disease), Wannan Medical College, Wuhu, Anhui China

**Keywords:** Difficult airway, Prediction, Geometry, Airway management, Computer

## Abstract

**Background:**

The anatomical characteristics of difficult airways can be analysed geometrically. This study aims to develop and validate a geometry-assisted difficult airway screening method (GADAS method) for difficult tracheal intubation.

**Methods:**

In the GADAS method, a geometric simulated model was established based on computer graphics. According to the law of deformation of the upper airway on laryngoscopy, the expected visibility of the glottis was calculated to simulate the real visibility on laryngoscopy. Validation of the new method: Approved by the Ethics Committee of Yijishan Hospital of Wannan Medical College. Adult patients who needed tracheal intubation under general anaesthesia for elective surgery were enrolled. The data of patients were input into the computer software to calculate the expected visibility of the glottis. The results of tracheal intubation were recorded by anaesthesiologists. The primary observation outcome was the screening performance of the expected visibility of the glottis for difficult tracheal intubation.

**Results:**

The geometric model and software of the GADAS method were successfully developed and are available for use. We successfully observed 2068 patients, of whom 56 patients had difficult intubation. The area under the receiver operating characteristic curve of low expected glottis visibility for predicting difficult laryngoscopy was 0.96 (95% confidence interval [CI]: 0.95–0.96). The sensitivity and specificity were 89.3% (95% CI: 78.1-96.0%) and 94.3% (95% CI: 93.2%-95.3), respectively.

**Conclusions:**

It is feasible to screen difficult-airway patients by applying computer techniques to simulate geometric changes in the upper airway.

**Supplementary Information:**

The online version contains supplementary material available at 10.1186/s12871-023-02312-9.

## Introduction

Difficulty in accurately screening difficult airways is a clinical pain point in airway management [1–5]. Most existing screening methods are based on the evaluation of anatomical landmarks of the body surface, so they have low accuracy [1, 2, 6–8]. The mechanism of the formation of difficult airways involves multiple factors [2, 9, 10]. The anatomy of the upper airway and the function of some joints are key factors [9–13], but the possible interactions between factors are unclear. Traditional difficult airway assessment methods can hardly reflect the interaction between these factors, and the contribution of these interactions to the formation of difficult airways is still unknown.

**Fig. 1:**
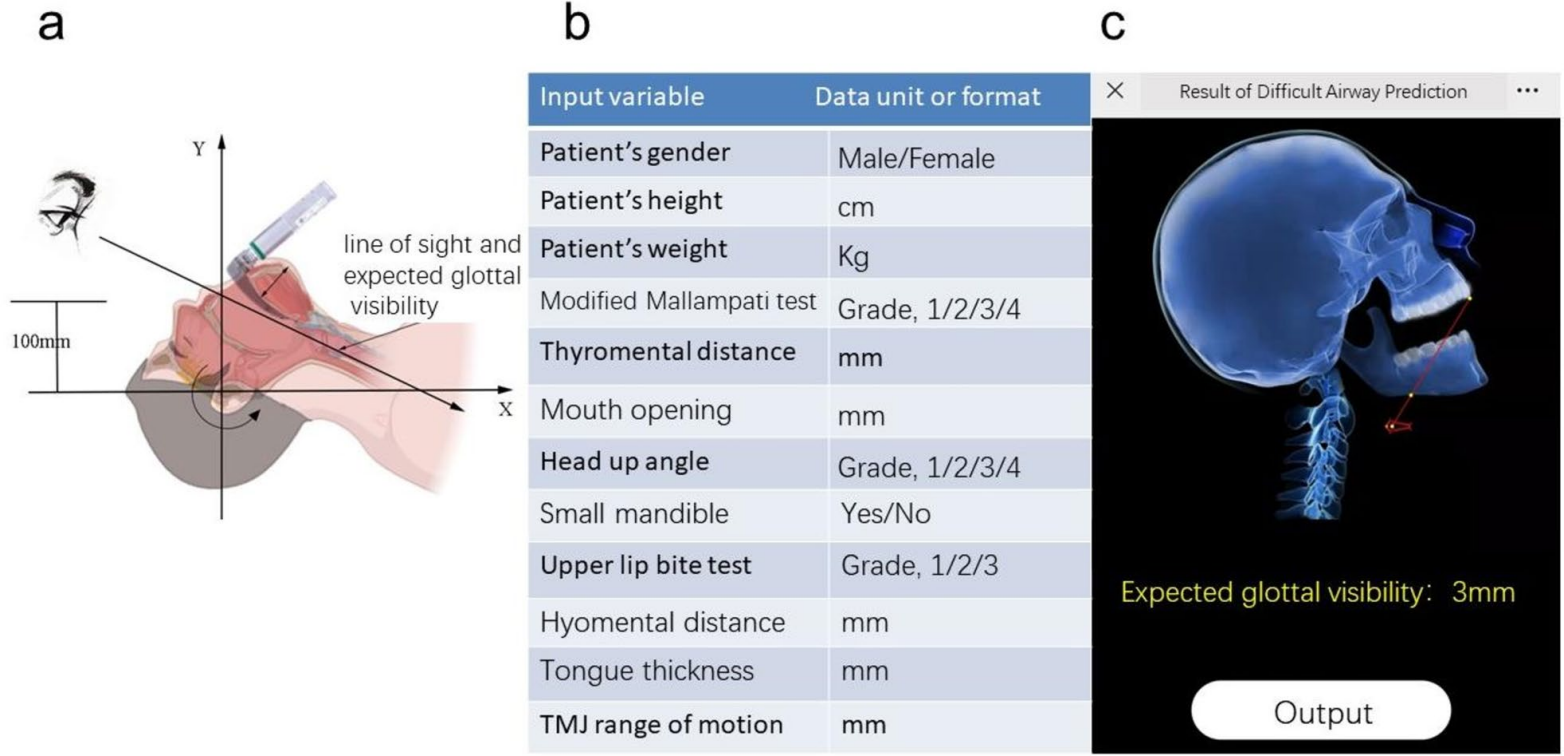
**a** Schematic diagram of geometric analysis of the upper airway structure; **b** Anatomical parameter or variable input interface (Supplementary Fig. 2: Chinese-English translation); **c** Result output, graphs and expected glottal visibility. Abbreviation: TMJ, temporomandibular joint

The composition of the upper airway seems to have geometric characteristics [14–17]. In brief (Fig. 1), the upper incisor, jaw, and upper pharynx form a fixed geometry whose positions are affected by the angle of the upper-back head. The mandible is linked with the tongue and hyoid bone and can be lifted by the laryngoscope in the forward and downward direction (relative to the patient; the same below), and the degree of displacement is limited by the range of mobility of the temporomandibular joint. The positions of the larynx and glottis are affected by the thyromental distance. Although a variety of multifactor combination scales are recommended for difficult airway screening [2, 18, 19], the simple summation of scores in the scale cannot reflect the geometric relationship between factors.

If we can use geometric figures to simulate these structures of the upper airway and simulate the changes and displacements of these structures during laryngoscopy, can we calculate the visibility of the glottis?

This study aimed to develop and validate a geometry-assisted difficult airway screening method (GADAS method), of which a digital graphics model was established to simulate the geometric changes in upper airway anatomy during laryngoscopy. The hypothesis was that by simulating the interaction between upper airway anatomy, joint function and other factors, the visibility of the glottis during laryngoscopy can be calculated, and difficult airways can be predicted.

## Materials and methods

### The establishment and application of the GADAS method

This part of the work was conducted by the research team together with mathematics experts and computer technology experts. Briefly, according to the upper airway anatomical characteristics and the methods of glottis laryngoscopy, a geometric model of the upper airway was established.

First, With reference to the approximate ratio of height and sagittal anatomical size of the head, as well as gender factors, a geometric simulation graphics library containing different sizes of the sagittal plane of the head and neck was established (Details are presented in Supplementary Material). We established the figures of the mandible, tongue, hyoid bone, and larynx graphics library separately. According to the patient’s body size (such as height and weight), the corresponding size of the head and neck sagittal geometry, as well as the geometry of the mandible, tongue, hyoid bone, and larynx were selected. With computer software, these graphics were manipulated in a two-dimensional plane, such as displacement, rotation, deformation, etc.

Second, we established the movement and deformation rules of the corresponding geometric figures during laryngoscopy. As shown in Fig. 1a, briefly, during laryngoscopy, the upper and lower axes of the head will deflect backwards. The mandible rotates clockwise to a produce mouth opening and is lifted and displaced by the laryngoscope in the forward and downward directions [13]. The tongue will be compressed, and the glottis will be revealed.

The head-up angle is defined as follows: When the patient is in the supine position and the head is in the head-up sniffing position, viewed from the side, the intersection of the vertical line passing through the earlobe with the face determines the grade of the head-up angle. Grade 1: The vertical line meets below the lower lip concavity. Grade 2: The vertical line intersects between the upper lip line and the lower lip concavity. Grade 3: The vertical line passes between the nose and the upper lip line. Grade 4: The vertical line passes through the nose (Supplementary Fig. 1). The rotational opening of the mandible is determined by mouth opening, measured by a ruler. The distance that the mandible moves forwards and downwards by laryngoscopy is determined by the degree of motion of the condyle of the temporomandibular joint [13]. Measurement of tongue thickness and retained thickness after compression to the original tongue thickness were based on previous research settings [11]. A small mandible was defined as a distance of < 4 cm from the lower incisor to the tip of the chin. The size of the larynx and glottis was set to a fixed value: the anteroposterior diameter of the larynx was 2.5 cm for males and 2.2 cm for females.

The position of the larynx and glottis is determined by the thyromental distance when the patient is in a head-up and sniffing position. The position of the hyoid bone is determined by the hyomental distance [20, 21].

**Fig. 2:**
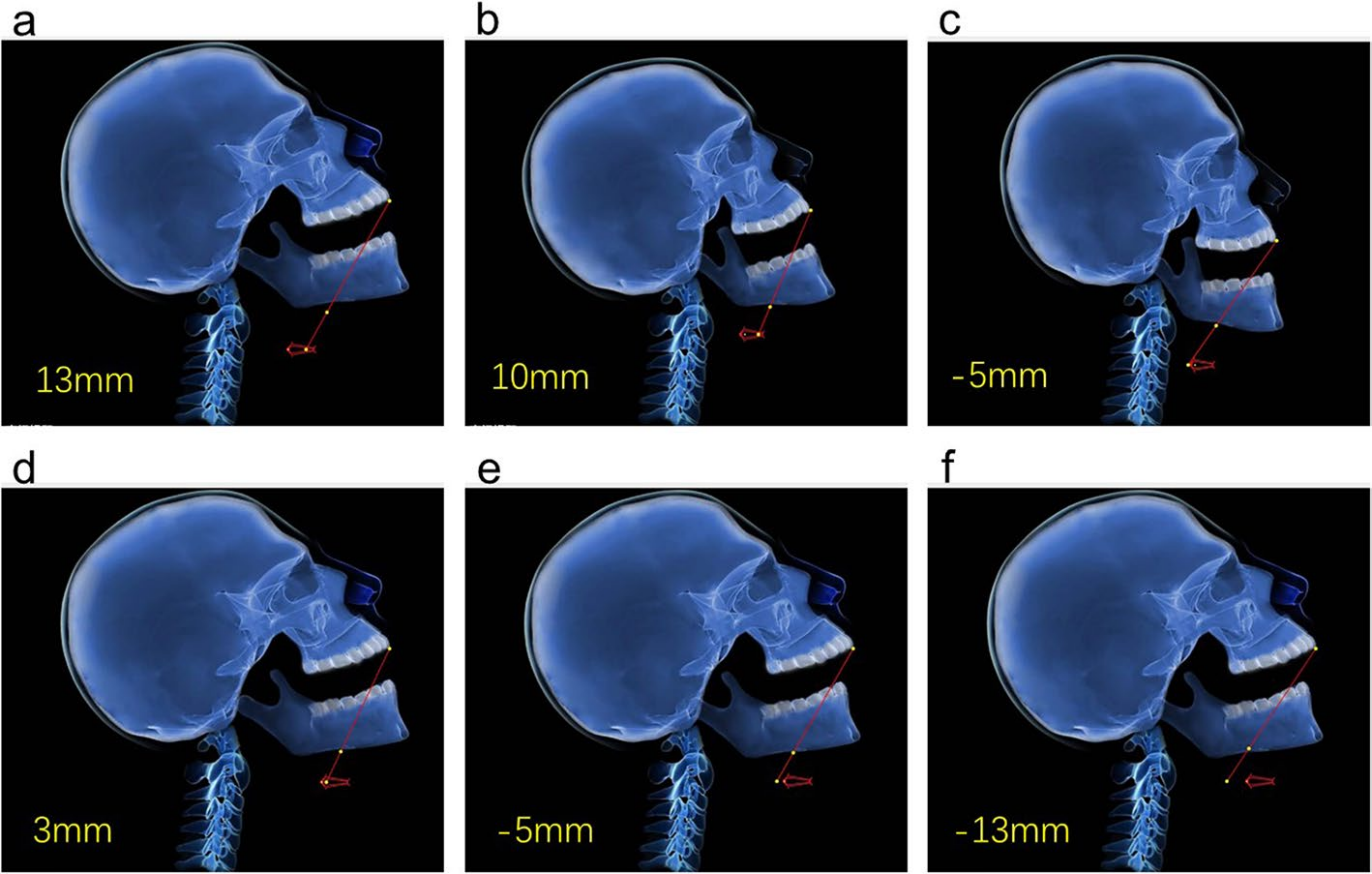
Examples of the change in input variables to the change in calculation results. All graphs are scaled the same. **a** to **b** height, 180 cm to 160 cm; **b** to **c** Head-up angle, grade 1 to grade 2; **a** to **d** thyromental distance, 9 cm to 7 cm; **d** to **e** TMJ range of motion 15 mm to 5 mm; **e** to **f** tongue thickness, 5.5 to 7 cm. Abbreviation: TMJ, temporomandibular joint

Using a computer software program, we simulated the displacement and deformation of the above-mentioned graphics in the two-dimensional plane and calculated the expected visibility of the glottis (Fig. 1b, c; and Supplementary Fig. 2). The change in the output figure caused by the input of different parameters is shown in Fig. 2.

The expected visibility of the glottis is defined as follows: The line of sight passes through the apex of the upper incisor, crosses the surface of the tongue, and extends to the glottis. If the line intersects the vocal cords, calculate the distance from the point of intersection to the posterior edge of the glottis and take a positive value. If the line cannot intersect the vocal cords, calculate the distance from the posterior edge of the glottis to the line of sight and take a negative value.

Overall structure of the software system: Information is input through an HTML5 web page, the corresponding information is transmitted to the central server through the network, the relevant program is called for calculation, and the result is returned to the terminal device through the HTML5 page (Fig. 1b, c; Supplementary Fig. 2).

### Verification of the GADAS method for screening difficult airways

After approval by the Ethics Committee of Yijishan Hospital of Wannan Medical College, a prospective observational case-cohort study was used to validate the new approach (ChiCTR-ROC-17013258, Registration date: 6th November 2017). Adult patients under general anaesthesia who required tracheal intubation for elective surgery were enrolled. Inclusion criteria: age over 18 years, no anatomical abnormalities of the head or face, and no airway stenosis or trauma. Exclusion criteria: known difficult airway, procedure cancellation, and missing data. Informed consent is required for enrolled patients. The input variables were sex, age, height, weight, mouth opening, modified Mallampati test, thyromental distance, head-up angle, tongue thickness, hyomental distance, temporomandibular joint range of motion, and small mandible. We entered the relevant variables on the information input page and recorded the output results.

Anesthesia induction was carried as follows: Midazolam (0.03 mg/kg), sufentanil (0.005 mg/kg), propofol (1–2 mg/kg), and rocuronium (1–1.5 mg/kg) were infused intravenously. Tracheal intubation was performed 3 min after the muscle relaxant injection.The responsible anesthesiologists (operator of the tracheal intubation, with more than 3 years of clinical anesthesia work experience. A total of 54 anesthesiologists were involved.) recorded the results of tracheal intubation, the visibility of the glottis and whether tracheal intubation was difficult. The visibility of the glottis during laryngoscopy was graded according to the modified Cormack and Lehane Grade [22, 23]. Grade 3 or 4 was classified as difficult laryngoscopy. Difficult tracheal intubation was defined as an experienced anaesthesiologist needing more than 2 intubation attempts, a total attempt time reaching 10 min, or the need to use advanced intubation equipment such as video laryngoscopes [10].

The primary outcome was the area under the receiver operating characteristic (ROC) curve (AUC) of the expected visibility of the glottis of the GADAS method to predict difficult tracheal intubation.

### Statistical analysis

Statistical analysis was performed using SPSS 18.0 (SPSS Inc., Chicago, IL, USA) and MedCalc 12.7 (Mariakerke, Belgium) software. The data of continuous variables are represented by the means and standard deviations, and the categorical variables are represented by frequencies and percentages. Statistical parameters are displayed as their value and their 95% confidence interval (95% CI). Two samples were compared by the independent-sample t test, nonparametric test or chi-square test, according to the specific situation. The ROC curve was drawn to analyse the performance of predictors in predicting difficult airways [24, 25]. The positive threshold was determined based on Youden’s index [24]. Binary logistic regression analysis was implemented using the “entry method. Taking *α* < 0.05, statistical power 0.8, AUC > 0.9, and significantly different from 0.8 (the common prediction performance of a single factor [8, 11]), based on a proportion of positive samples of 2.5% [11], the calculated sample size needed was > 2000.

## Results

### Geometric model establishment and computer software development of the GADAS method

The research team developed usable software that followed the design ideas and plans. The model of the upper airway–related anatomy and the calculation function of the software were obtained. It took approximately 90 s for the user to input the required information about a patient. With the support of a 4G mobile network, it took approximately 4 s to return the calculation result. The use and calculation efficiency were in line with the original intention of the design (Software services can be obtained through WeChat’s public service account: “爱气道 AI Airway”).

### Validation of the performance of difficult airway screening

#### Patient information

**Fig. 3:**
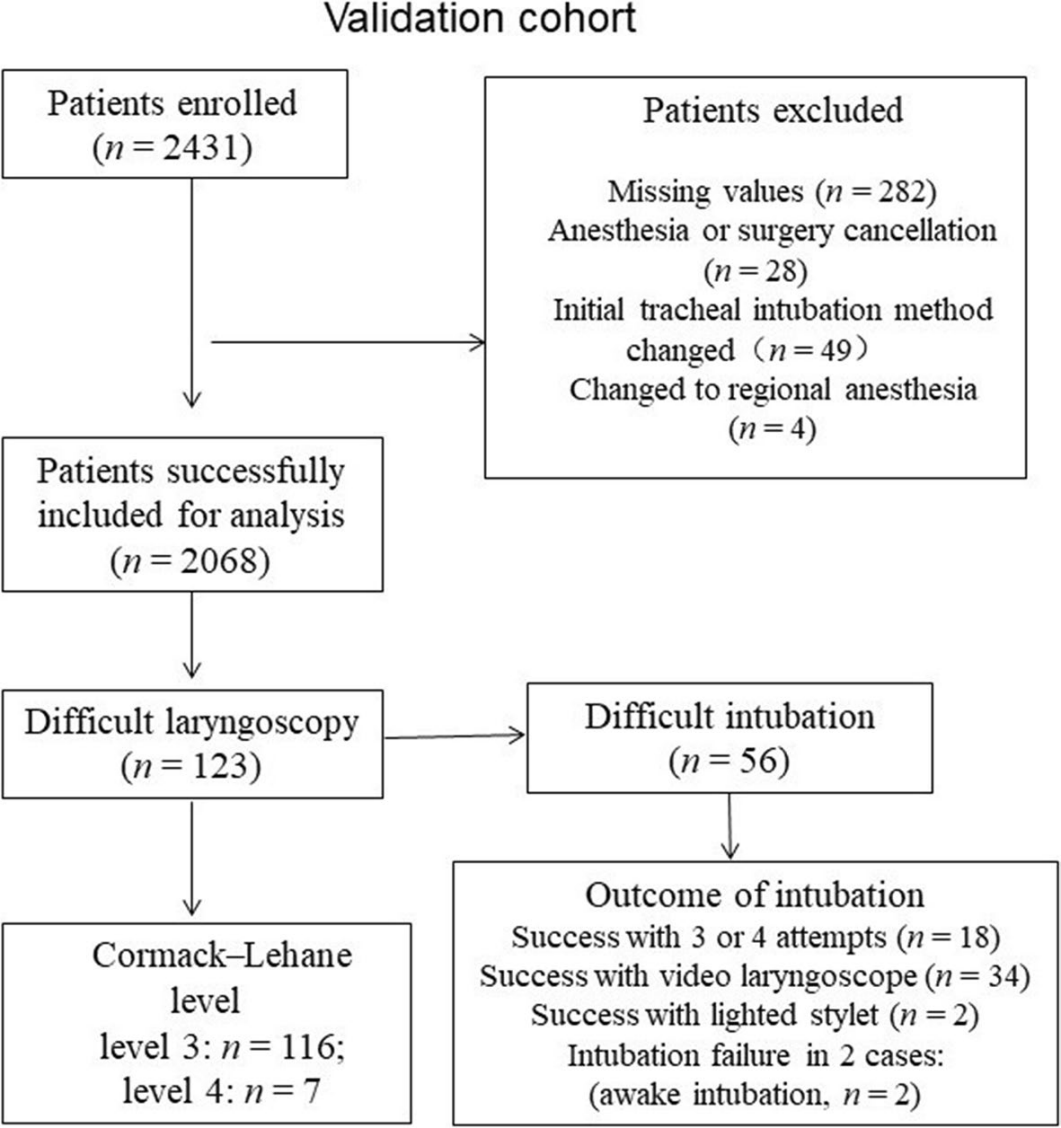
The study flow chart

Computer-calculated results were obtained from 2068 enrolled patients, including 1001 males and 1067 females. In total, 123 cases had difficulty with laryngoscopy, and 56 cases had difficulty with intubation. For details, see the flowchart (Fig. 3). The characteristic data and expected glottal visibilities of the patients are shown in Table 1. The expected glottal visibilities were significantly different in patients with vs. without difficult intubation and with vs. without difficult laryngoscopy (*P* < 0.001).

**Table 1:** Comparison of variables between patients with and without difficult airways (*n* = 2068)

Variable	Difficult intubation			Difficult laryngoscopy		
	Yes (*n* = 56)	No (*n* = 2012)	*P* Value*	Yes (*n* = 123)	No (*n* = 1945)	*P* Value*
Sex, *n*			0.185			0.001
Male/	32	969		78	923	
Female	24	1043		45	1022	
Age, yr, median (IQR)	62 (52–69)	50 (41–62)	< 0.001	61 (50–67)	50 (40–61)	< 0.001
Body mass index, kg/m^2^	23.4 (3.6)	22.9 (3.5)	0.260	23.3 (3.4)	22.8 (3.5)	0.134
Thyromental distance, cm	6.7 (0.8)	7.7 (0.9)	< 0.001	7.0 (0.9)	7.7 (0.9)	< 0.001
Modified Mallampati test > 2, *n* (%)	34 (60.7)	537 (26.7)	< 0.001	61 (49.6)	510 (26.2)	< 0.001
Mouth opening, cm	3.4 (0.5)	4.1 (0.6)	< 0.001	3.6 (0.6)	4.1 (0.6)	< 0.001
Hyomental distance, cm	4.6 (0.4)	5.3 (0.5)	< 0.001	4.7 (0.5)	5.3 (0.5)	< 0.001
Tongue thickness, cm	6.3 (0.5)	5.9 (0.5)	< 0.001	6.3 (0.5)	5.9 (0.5)	< 0.001
Grade of head-up angle, 1/2/3/4, n	30/26/0/0	2006/6/0/0	< 0.001	94/29/0/0	1942/3/0/0	< 0.001
Small mandible, yes, n (%)	15 (26.8)	51 (2.5)	< 0.001	23 (18.7)	43 (2.2)	< 0.001
TMJ range of motion, mm	9.5 (2.5)	14.0 (2.3)	< 0.001	10.7 (2.7)	14.1 (2.2)	< 0.001
Expected glottal visibility, mm	-11.0 (8.1)	9.6 (8.3)	< 0.001	-5.3 (9.8)	9.9 (8.1)	< 0.001

Ages are presented as median (interquartile ranges: 25th percentile, 75th percentile); other continuous variables are represented as the mean (standard deviation)

*Abbreviation*: *TMJ* Temporomandibular joint

*P value tested by the independent-sample *t* test, nonparametric test or chi-square test, according to the specific situation

#### Prediction of difficult airways

**Fig. 4:**
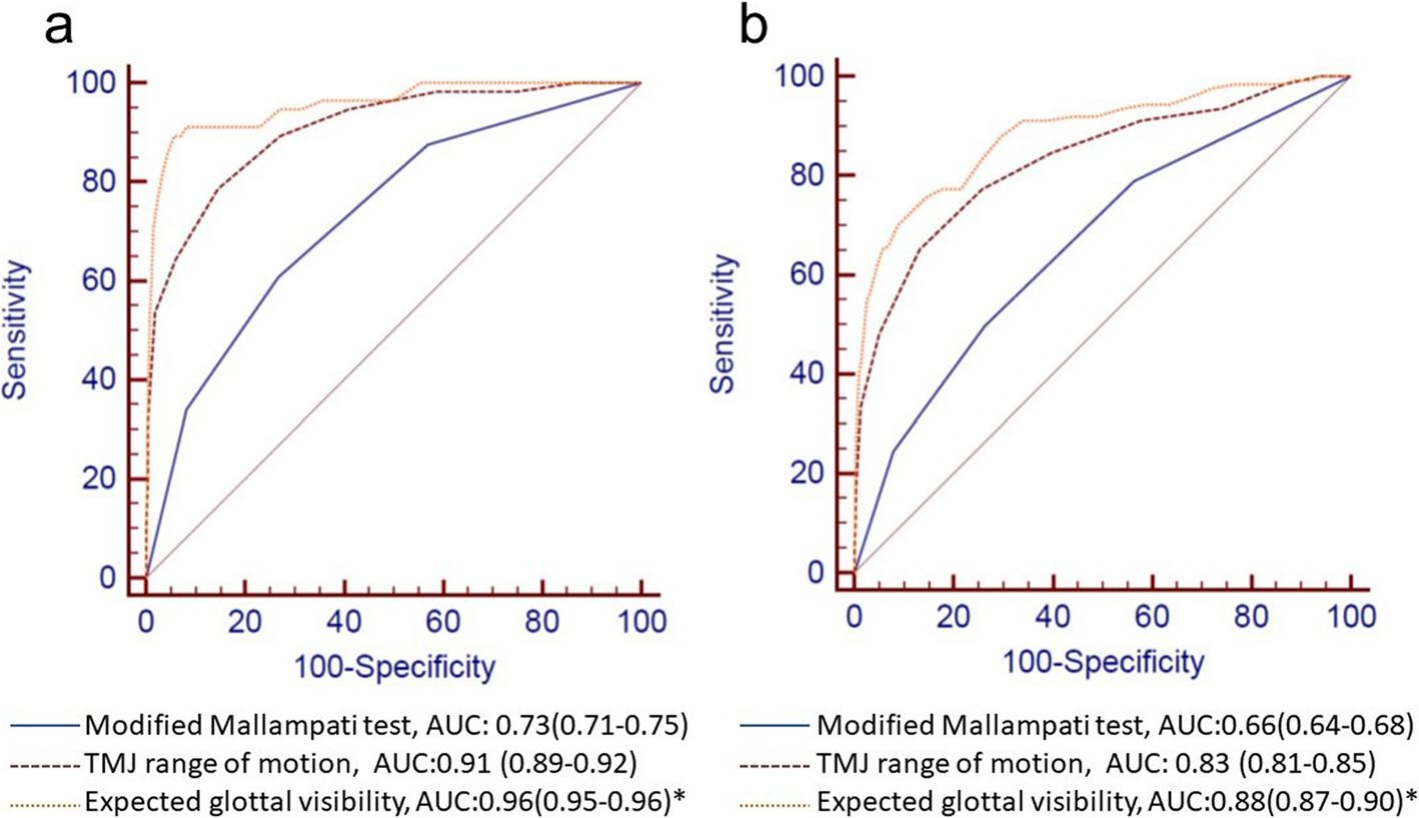
ROC curve analysis of independent risk factors for predicting difficult intubation (**a**) and difficult laryngoscopy (**b**). At the bottom are the values of AUCs and their comparisons. *: Statistically significant difference from others. Abbreviation: TMJ, temporomandibular joint; AUC, area under the curve; ROC, receiver operating characteristic curve

ROC curve analysis showed that the AUC of expected glottal visibility in predicting difficult intubation was 0.956 (95% CI: 0.946 to 0.964). Under the positive standard of ≤ -4 mm, the sensitivity was 89.3% (95% CI: 78.1–96.0%) and the specificity was 94.3% (95% CI: 93.2–95.3%). After logistic regression analysis, the remaining independent factors were the modified Mallampati test result, TMJ range of motion, and expected glottal visibility. The corresponding ROC curves, AUCs and diagnostic parameters of independent factors are shown in Fig. 4; Table 2. The AUC of low expected glottal visibility was significantly higher than that of other difficult-airway predictors (*P* < 0.05).

**Table 2:** Predictive values of independent predictors of difficult intubation and difficult laryngoscopy (*n* = 2068)

Variable (Positive threshold value)	Odds ratio (95% CI)	Sensitivity (95% CI)	Specificity (95% CI)
For difficult intubation			
Modified Mallampati test (> 2)	4.24 (2.46–7.32)	0.61 (0.47–0.74)	0.73 (0.71–0.75)
TMJ range of motion (≤ 11 mm)	21.69 (11.32–41.55)	0.79 (0.66–0.88)	0.86 (0.84–0.87)
Expected glottal visibility (≤-4 mm)	138.74 (58.26-330.39)	0.89 (0.78–0.96)	0.94 (0.93–0.95)
For difficult laryngoscopy			
Modified Mallampati test > 2	2.77 (1.92-3.00)	0.50 (0.41–0.59)	0.74 (0.72–0.76)
TMJ range of motion (≤ 11 mm)	12.33 (8.32–18.27)	0.65 (0.56–0.73)	0.87 (0.85–0.88)
Expected glottal visibility (≤-1 mm)	24.43 (16.11–37.04)	0.70 (0.61–0.78)	0.91 (0.90–0.93)

*Abbreviations*: *CI* Confidence interval, *TMJ* Temporomandibular joint

For the prediction of difficult laryngoscopy, the AUC of the expected glottal visibility was 0.883 (95% CI: 0.868–0.897). The sensitivity and specificity were 69.2% (95% CI: 61.0–77.9%) and 91.3% (95% CI: 90.0–92.5%), respectively (under the positive standard of ≤ -1 mm). The corresponding diagnostic parameters of independent predictors are shown in Fig. 4; Table 2. Similarly, compared with other independent predictors of difficult airways, low expected glottal visibility had a significantly higher AUC (*P* < 0.05).

## Discussion

Our work shows that it is feasible in the GADAS method to apply the geometric simulation method based on computer technology to the study of difficult airways. This method can well simulate the formation of difficult airways. Our research also shows the excellent performance of this method when used to predict difficult airways, better than the single-factor predictive performance of difficult airways. Unlike previous authors [14–17], we did not apply the technology of 3-dimensional simulation, which would simplify the algorithm and reduce the design difficulty. Whether applying 3-dimensional technology can increase difficult airway screening performance is unclear.

The screening performance of difficult airways using a single factor is poor [6–8]. The formation of difficult airways is determined by many factors [2, 9], some of which are known and some still unknown. Even among these known factors, understanding the interactions between them is difficult. For example, what happens when the limited range of motion of the head or neck and the limited range of motion of the temporomandibular joint exist at the same time? We tried to apply geometric methods to simulate this process. We believe that our method can reflect the geometric interaction between different factors to a certain extent (Fig. 2). The good predictive performance of difficult airways shows the effectiveness of our method.

In many studies of difficult airways involving multiple factors, linear regression analysis or logistic regression analysis is often used to solve the problem of multifactor interactions [11, 26]. When solving problems, the regression analytical method itself has difficulty answering questions, such as, is the interaction between these factors linear? How can we use the independent risk factors selected by regression analysis? If analysed from the perspective of geometry, we find that the interaction of many factors may not be linear. For example, the angle of head up, the angle of rotation of the mandible when opening the mouth, the position of the larynx, etc., there are several trigonometric relationships between these factors, and their interaction is complicated. It is also difficult to calculate the interaction between them by deriving mathematical formulas, as well as to visualize the glottis. The complexity of this work is far greater than that of the application of regression equations.

Our approach effectively avoided this difficulty. We applied the method of equal-size graphic simulation to reproduce the changes and displacements of various factors during laryngoscopy and finally applied the method of connecting the target points to simulate the reach of the line of sight. We have omitted many formula derivation, verification and calculation processes and simplified the calculations. However, whether this method has advantages in accuracy and time efficiency compared with methods that also apply multi-factor scoring (e.g., El Ganzouri-score) requires further research.

This study has some limitations. Among the many factors causing difficult airways, we only included some of them and used them in a way that we could understand. This understanding is based on the anatomical displacement and deformation rules of the upper airway. There are some shortcomings of doing so. For example, some variables are difficult to incorporate into the geometric model because the mechanism involved in the geometric deformation of the upper airway is unknown, such as neck circumference [27, 28]. If we can learn the participation mechanisms of more factors and simulate them, the results should be more accurate. Discovering more sensitive factors and understanding the mechanism of their participation in difficult airways should be a direction of future research. Although the incidence of difficult airways varies among patients of different specialties, it is difficult to conduct subgroup analysis for them in this study. Prospective clinical trials involving patients in different age ranges and in different clinical scenarios are needed.

In summary, Based on geometry simulation, we have developed a new kind of difficult airway screening method— the GADAS method. Our preliminary data show the effectiveness of this method. As methods improve, the screening performance should further improve.

### Supplementary Information

**Additional file 1: Supplementary Fig 1.** Grading of the head-up angle. **Supplementary Fig 2.** Software input and output.

## Data Availability

The datasets used and/or analysed during the current study available from the corresponding author on reasonable request.
