# Supplementary material for: Development and validation of a screening method for difficult tracheal intubation based on geometric simulation and computer technology
Source: BMC Anesthesiol. 2023 Oct 25;23:350. doi: 10.1186/s12871-023-02312-9 (PMC10598895; doi:10.1186/s12871-023-02312-9)
Supplement: Supplementary file 1 — Additional file 1: Supplementary Fig 1. Grading of the head-up angle. Supplementary Fig 2. Software input and output. [file 12871_2023_2312_MOESM1_ESM.docx]

Supplementary Material

Brief introduction to the implementation steps of airway assessment geometry simulation

1. Establishment of the Sagittal Anatomical Schematic Library of the Head and Neck

Draw the schematic diagrams of the geometric projection simulation of the sagittal plane of the head and neck with different sizes. Among them, the upper jaw and the skull are integrated; the mandible, cervical spine, hyoid bone, tongue, larynx and glottis are drawn separately. The graphic size gradient interval is 1mm. The graphic size range meets the selection of patients whose height is between 130cm and 200cm.

2. Canvas coordinate setting and appropriate graphic selection and layout.

Set up a two-dimensional coordinate system with the ear holes of the skull as the origin, the downward direction of the body as the X-axis, and the forward direction as the Y-axis. According to the proportional relationship between height and head and neck size, select the head and neck simulation image of the corresponding size of the patient. According to the size classification of the mandible, select the size type of the mandible. Set the position of the larynx according to the thyromental distance. Set the position of the hyoid bone according to the hyomental distance. Set the position of the vertices of the tongue according to the thickness of the tongue., etc.

3. According to the characteristics of movement of the head, neck and mandible during the laryngoscope examination of the glottis, the simulation of graphic movement and deformation is carried out.

According to the classification of the patient's head-up angle, set the head-up angle of the skull figure during laryngoscopy. The position of the mandible during laryngoscopy is set according to the degree of mouth opening and the range of motion of the temporomandibular joint. Refer to the position of the mandible after displacement, set the position of the larynx according to the thyromental distance; set the position of the hyoid bone according to the hyomental distance; and set the position of the vertices of the tongue according to the thickness of the tongue, the position of the hyoid, and the compression ratio of the thickness of the tongue.

4. Determine the direction of the line of sight and the size of the visible glottis according to the positional relationship after the graphic movement.

Referring to the simulated deformed image, connect the upper incisor-vertice of the tongue, and extend to the glottis level. The expected visible glottis is calculated from the intersection of the connecting line with the glottis. The visible glottis is defined as the distance from the intersection point of the connecting line to the posterior edge of the glottis. If the intersection point is in front of the posterior border of the glottis, the value is positive, meaning that the size of the glottis can be seen. If the intersection point is behind the posterior edge of the glottis, its value is negative, which means the distance between the line of sight and the glottis.


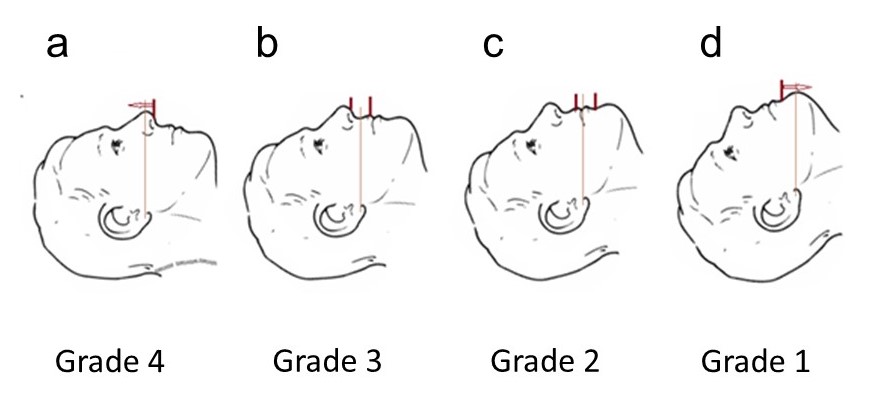


Supplementary Fig 1. grading of the head-up angle.

When the patient is in the supine position and the head is in the head-up sniffing position, viewed from the side, the intersection of the vertical line passing through the earlobe with the face determines the grade of the head-up angle. a: Grade 4, the vertical line passes through the nose; b: Grade 3, the vertical line passes between the nose and the upper lip line; c: Grade 2, the vertical line intersects between the upper lip line and the lower lip concavity; d: Grade 1, the vertical line meets below the lower lip concavity.


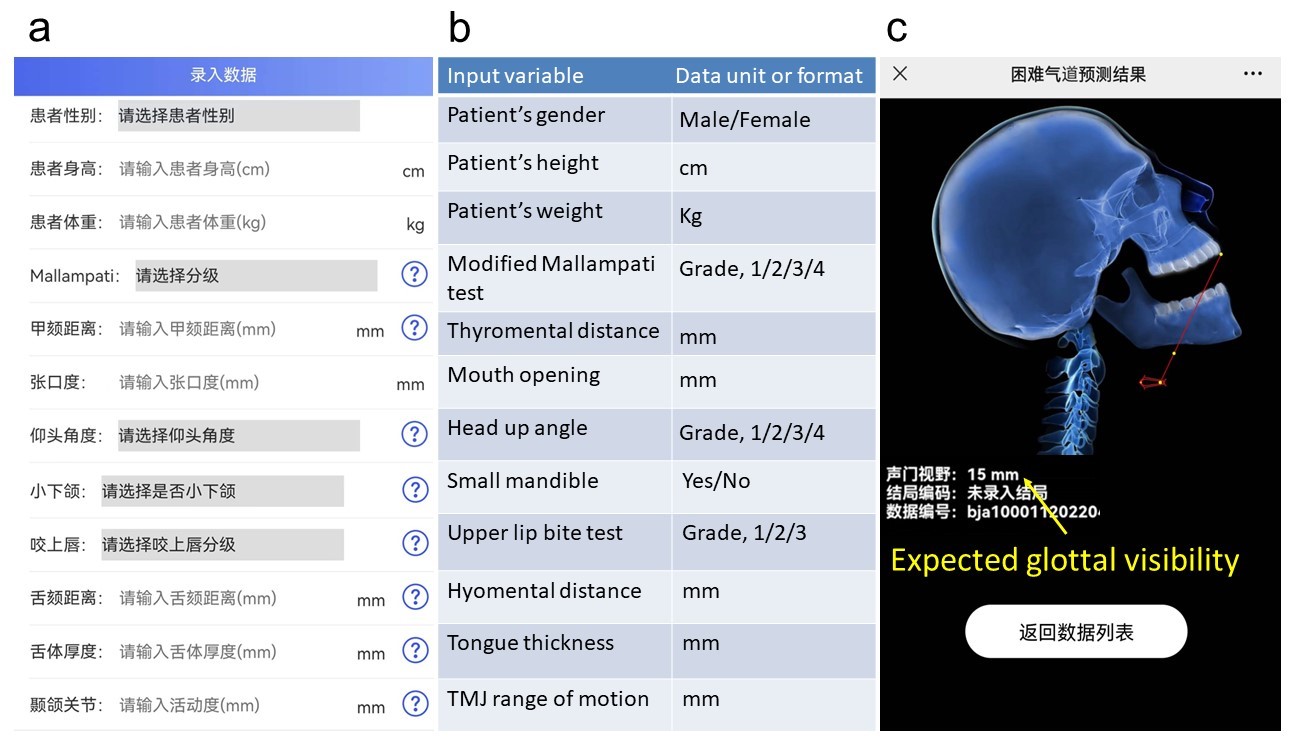


Supplementary Fig 2. Software input and output.

a: Input parameters of the software (Chinese form). b: Corresponding English translation. c: Graphics and calculation results output by the software.
